# 3D-Ultrasound Based Mechanical and Geometrical Analysis of Abdominal Aortic Aneurysms and Relationship to Growth

**DOI:** 10.1007/s10439-023-03301-2

**Published:** 2023-07-06

**Authors:** Esther Jorien Maas, Arjet Helena Margaretha Nievergeld, Judith Helena Cornelia Fonken, Mirunalini Thirugnanasambandam, Marc Rodolph Henricus Maria van Sambeek, Richard Godfried Paulus Lopata

**Affiliations:** 1 PULS/e Group, Department of Biomedical Engineering, Eindhoven University of Technology, Eindhoven, The Netherlands; 2 Department of Vascular Surgery, Catharina Hospital Eindhoven, Eindhoven, The Netherlands

**Keywords:** AAA, Arterial compliance, Volume, Curvature, Growth prediction, Time-resolved 3D ultrasound

## Abstract

The heterogeneity of progression of abdominal aortic aneurysms (AAAs) is not well understood. This study investigates which geometrical and mechanical factors, determined using time-resolved 3D ultrasound (3D + t US), correlate with increased growth of the aneurysm. The AAA diameter, volume, wall curvature, distensibility, and compliance in the maximal diameter region were determined automatically from 3D + t echograms of 167 patients. Due to limitations in the field-of-view and visibility of aortic pulsation, measurements of the volume, compliance of a 60 mm long region and the distensibility were possible for 78, 67, and 122 patients, respectively. Validation of the geometrical parameters with CT showed high similarity, with a median similarity index of 0.92 and root-mean-square error (RMSE) of diameters of 3.5 mm. Investigation of Spearman correlation between parameters showed that the elasticity of the aneurysms decreases slightly with diameter (p = 0.034) and decreases significantly with mean arterial pressure (p < 0.0001). The growth of a AAA is significantly related to its diameter, volume, compliance, and surface curvature (p < 0.002). Investigation of a linear growth model showed that compliance is the best predictor for upcoming AAA growth (RMSE 1.70 mm/year). To conclude, mechanical and geometrical parameters of the maximally dilated region of AAAs can automatically and accurately be determined from 3D + t echograms. With this, a prediction can be made about the upcoming AAA growth. This is a step towards more patient-specific characterization of AAAs, leading to better predictability of the progression of the disease and, eventually, improved clinical decision making about the treatment of AAAs.

## Introduction

In this study, we present a fully ultrasound-based and automated analysis of the 3D geometrical and mechanical parameters of abdominal aortic aneurysms in a large group of patients (N = 172). The relationship between these parameters and the size and growth of aneurysms is investigated and compared to current clinical standards.

An abdominal aortic aneurysm (AAA) is a local dilatation of the aorta, which usually grows over time. The larger the aneurysm becomes, the higher the rupture risk [1], leading to a life-threatening and often fatal hemorrhage. To prevent this, surgical repair is performed when the risk of rupture is deemed to be higher than the risks associated with intervention. Therefore, AAA diameters are regularly monitored in the outpatient clinic using ultrasound, and surgery is performed when a diameter of 5.5 or 5.0 cm is reached for men and women, respectively, or the AAA grows more than 1 cm/year [2].

There is however quite some heterogeneity in the progression of the disease, with some AAAs showing no growth for several years, or even shrinkage, while other AAAs grow as much as 1.3 cm/year [3–5]. Furthermore, there is variation in the diameter at which rupture occurs between patients: some smaller abdominal aneurysms of less than 5 cm rupture [6], while other larger aneurysms stay stable, even up to diameters of 20 cm [7, 8]. These differences in growth and risk of rupture might be related to differences in the way that the aortic wall adapts to the changes in wall deformation as well as the load (pressure, blood flow pattern) it experiences while the AAA grows [9, 10]. There is a wide variety of abdominal aneurysm geometries known to develop in the human body [11]. The mechanical properties of AAAs show large variations as well, with in vivo pressure-strain elastic moduli of 0.55–9.46 Pa [12], and aortic stiffnesses of 0.8–4.5 kPa m [13].

Previous studies investigating the disparity in AAA growth rates have shown the dependence of growth on lifestyle and presence of comorbidities such as smoking, hypertension and diabetes [4, 14, 15]. Furthermore, the relation between growth rate and diameter has been well established [3, 15, 16].

More recently, studies have been performed on using medical imaging techniques to find parameters related to AAA diameter and growth. Computed tomography (CT) has been used to study relationships between AAA geometry and growth: a study by Chandrashekar et al. [17] showed the predictive value of curvature for the upcoming AAA growth, and Lindquist Liljeqvist et al. [18] showed the benefit of volume as a growth predictor over the diameter. Furthermore, the merit of combining multiple features of the AAA for growth prediction has been shown [19]. However, an important disadvantage of CT is the use of ionizing radiation, which prevents it from being used in frequent follow-up studies to monitor the AAA.

The dynamic properties of AAAs have been studied using magnetic resonance imaging (MRI) and ultrasound (US). An MRI-based study found no correlation between AAA stiffness and diameter [20]. US-based studies on mechanical changes of the aortic wall with aneurysm progression resulted in contradicting findings: Wilson et al. [12] showed an increase in elastic modulus with diameter, while Long et al [21] showed an increased compliance with diameter. MRI imaging is however expensive, making it unsuitable for follow-up studies, and standard US is only 2-dimensional (2D), lacking information to capture the complex 3-dimensional (3D) shapes of AAAs.

With time-resolved 3D ultrasound (3D + t US), it is possible to capture both the full 3D geometry and the motion of the AAA during the cardiac cycle, while avoiding the use of radiation and at relatively low cost. Previous studies using 3D + t US have shown that it allows for the calculation of strain patterns [22] and volume of AAA [23, 24]. Furthermore, a combination of 3D + t US with a finite- element updating approach enables estimating the stiffness [25, 26].

Despite the merits of these efforts, a cost-effective and non-invasive method for consecutively determining the diameter, volume, curvature, and elasticity of the AAA is still lacking. This study aims to develop and validate a method that enables automatic and direct estimation of both geometrical and mechanical parameters of AAAs using 3D + t US. Furthermore, it aims to apply this method to a large patient cohort, to study mutual relationships between these parameters, as well as relation to aneurysm growth.

## Materials and Methods

### Data Acquisition

3D + t echograms of AAAs were acquired by experienced sonographers at the Catharina hospital in Eindhoven, the Netherlands, for 172 patients, during routine diameter check-ups between 2014 and 2022. This study was approved by the local ethics committee of the Catharina Hospital Eindhoven, and all patients gave written informed consent. Either a Philips iU22 or EPIQ ultrasound system was used, both equipped with an X6-1 matrix probe with a 3.5 MHz center frequency, resulting in volume rates of 3.2–7.5 Hz. All 3.0–7.6 seconds long acquisitions were performed in supine position during breath hold, after which the blood pressure was measured using an arm cuff. Patients’ age and self-reported gender were noted. Furthermore, 2D ultrasound-based diameter measurements (*d*_2*D*_) of the aorta were obtained for the current and each follow-up visit of the patient. Inclusion criteria were at least 2 follow-up diameter measurements and sufficient echogenicity to visually see the AAA in the 3D + t US images. All anonymized data were processed in MATLAB R2021a.

A subgroup of patients received a CT scan as part of their routine clinical care. All CTs acquired within 2 months of a 3D + t echogram were collected for validation of the US-derived geometrical parameters. Since most CTs were performed when the threshold diameter for surgery was met, growth analysis after this timepoint was not possible. Hence, for some patients, two 3D + t echograms from different dates were used: one for comparison to CT, and the other for growth analysis.

### Image Processing

**Fig. 1:**
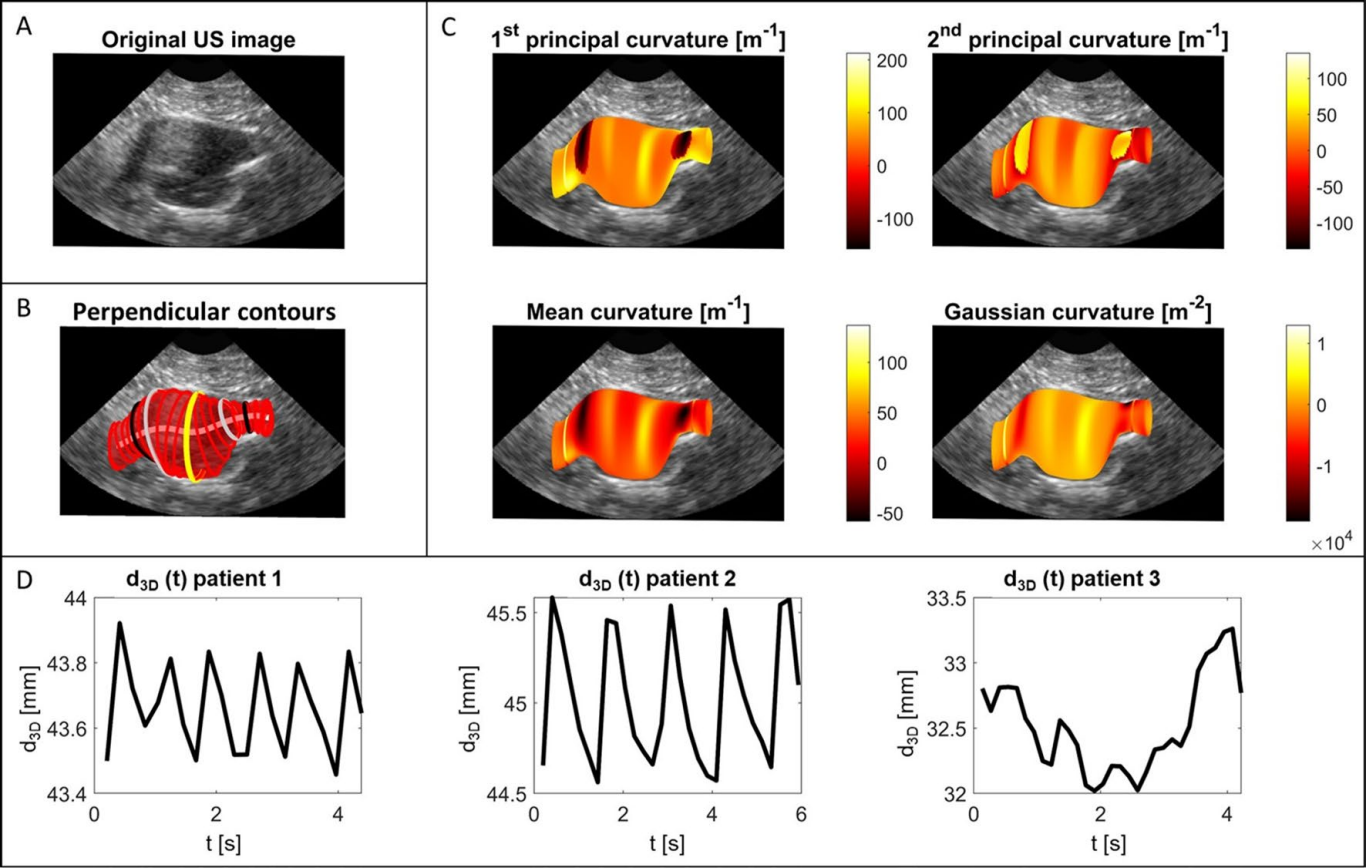
Illustration of the acquired geometrical and mechanical parameters, showing **A** a longitudinal slice of the original ultrasound image, **B** the perpendicular contours to the centerline (only every 5 mm for visual purposes) with the maximal diameter contour in yellow, edges of the 60 mm volume in black, and edges of the 40 mm volume in grey, **C** the different curvature parameters that were determined and **D** examples of the d_3D_ over time graphs for three patients: the first two with clear and relatively constant pulsations, and a third one with non-constant pulsation height

The 3D geometry of the aortic wall was obtained by segmenting all time frames (n = 14–43) of the 3D + t US data using an in-house developed fully automatic algorithm. In summary, an ellipse is fitted to transverse slices of the 3D volume [27] and updated on each slice using a Star-Kalman based algorithm [28, 29], followed by 3D active deformable contours to improve the accuracy and continuity of the AAA geometry [30]. The segmentation results were visually checked by plotting onto the data (Fig. 1A, B), and where needed, the proximal and distal ends of the geometry were manually cut off.

### Geometric Parameters

An average of the geometries derived from all the frames was determined, and its centerline was extracted based on a Dijkstra shortest path algorithm [31–33]. Contours perpendicular to this centerline were created (Fig. 1B), from which the location and magnitude of the maximal diameter (*d*_3*D*_) were calculated. The partial volumes *V*_*part,L*_ of the AAA were determined in a region with a centerline path length (*L*) of 30, 40, 50, 60 and 70 mm around *d*_3*D*_*.*

Furthermore, the local principal curvatures *K*1 and *K*2 of the inner vessel wall were determined using a finite-differences approximation [34, 35] in the complete region visible in the US image (Fig. 1C). These local principal curvatures are closely related to the wall stress [36], indicating regions at risk and in which changes in the aortic wall are expected to happen. From the local principal curvatures, the point-wise mean (*M*) and Gaussian curvatures (*G*) were determined by respectively averaging and multiplying the local values of *K*1 and *K*2. The sign of *M* indicates whether the region is locally more convex (+) or concave (−), while a negative value of *K* indicates a saddle point. For all curvature parameters *X*, their area-average (*XAA*) and L2-norm (*XLN*), a measure of irregularity, were determined [11].

### Mechanical Parameters

The average anterior-posterior diameter was determined for each time frame in a 30 mm long region around maximal diameter, in which only the ventral and dorsal sides of the geometry were incorporated because of best image quality. The resulting diameter-time curves (Fig. 1D) were manually checked for clear peaks with constant prominence, from which distension could reliably be determined. The distensibility *D* of the vessel was calculated using the automatically selected systolic and diastolic diameters, *d*_*sys*_ and *d*_*dia*_, and pulse pressure ∆*p*, according to

$$D=\frac{{d}_{sys}^{2}-{d}_{dia}^{2}}{{d}_{dia}^{2}\cdot\Delta p}$$

Furthermore, the compliance *C* in a volume of length *L* was determined as

$${C}_{L}=\frac{\Delta {V}_{part,L}}{\Delta p}\approx \frac{{V}_{part,L}\cdot \frac{{d}_{sys}^{2}-{d}_{dia}^{2}}{{d}_{dia}^{2}}}{\Delta p}={V}_{part,L}\cdot D,$$

with ∆*V*_*part,L*_ the diastolic-to-systolic volume change in *V*_*part,L.*_ This formulation determines the small displacements from regions with best US contrast, as was done for the diameters (ventral and dorsal sides in the middle of the 3D volume).

### Validation of Geometric Parameters with CT

The available CT scans were segmented using the semi-automatic segmentation package Hemodyn, developed by Philips Medical Systems (Best, The Netherlands) and Eindhoven University of Technology (TU/e). The resulting geometries were matched with the US-based geometries by aligning the centerlines and maximal diameters, and then finetuning the alignment with an iterative closest point algorithm [37].

The similarity of the US and CT-based geometries in the overlapping region was quantified using the similarity index (*SI*) and the 95th-percentile distance (*D*95), an alternative to the Hausdorff distance less sensitive to outliers [38]. Furthermore, CT-derived geometrical parameters (diameter d_CT_, volume and curvature) were determined with the same methods applied to the US geometry. The *d*_*CT*_ was compared to both *d*_3*D*_ and *d*_2*D*_, to compare accuracy of 3D US-based diameters with the clinically used 2D US-based diameter. The US and CT curvature parameters were compared with Spearman’s rank correlation.

### Statistical Analysis

Linear regression of all (*≥* 3) diameter values over time was performed, and its slope was regarded as the growth after the datapoint of interest.

In a first exploratory analysis, the correlation between all geometrical, mechanical and demographic parameters was investigated using a Spearman’s rank correlation. A p-value of 0.05 was seen as a statistically significant difference. Bonferroni-adjusted p-values (p < 0.002) are mentioned separately. All parameter values are indicated as median [IQR].

Next, parameters of interest were corrected for their correlation with diameter by subtraction with the regression line, and Spearman’s correlations with other parameters were redetermined. Furthermore, relationships of interest were further investigated by splitting the independent variable in three groups, and comparing the groups with a Wilcoxon rank sum test. To ensure comparable group sizes for statistical analysis, the boundaries of the groups were set to the 33rd and 67th percentile values of the independent variable, resulting in groups of < 36 mm, 36–43 mm and > 43 mm for maximal diameter and < 100 mmHg, 100–108 mmHg and > 108 mmHg for mean arterial pressure.

Finally, the possibility of determining a linear growth prediction model was investigated. The relevant parameters were selected using step-wise regression. The performance of the best growth model was evaluated with the root mean squared error (RMSE), and compared to a growth prediction based on the diameter.

## Results

### Algorithm Performance and Outcomes

An overview of the patient demographics is shown in Table 1. The automatic segmentation algorithm was successful in 167 out of 172 patients. The volumes, distensibility, and compliances could only be determined for a subgroup of patients (indicated in Table 2), with *V*_*part,L*_ limited by the field of view, *D* by the lack of clear and constant pulsations in the diameter-time curves, and *C*_*L*_ by both.

**Table 1:** Patient demographics and follow-up information, reported in [median (range)]

Variable	Value
Gender (M:F)	143:29
Age (years)	75 (53–90)
Max. clinical diameter at inclusion (mm)	43 (29–59)
Diastolic blood pressure *p*_*dia*_ (mmHg)	84 (56–118)
Systolic blood pressure *p*_*sys*_ (mmHg)	144 (93–197)
Number of follow-up visits (−)	5 (3–20)
Follow-up time (months)	39 (6–84)
Growth rate (mm/year)	1.8 (− 1.9 to 9.1)

**Table 2:** Values of the parameters determined, and the number of patients for which this parameter could be determined. d_2D_ is the diameter from 2D ultrasound, d_3D_ the diameter perpendicular to the centerline from 3D ultrasound, and V_part,L_ the volume in a L mm long region around maximal diameter. [X]AA and [X]LN are the area-average and L2-norm of the first and second principal curvatures K1 and K2, and the mean and Gaussian curvatures K and G. D is the distensibility, and C_L_ is the compliance in a L mm long region around d_3D_

Variable	Median (IQR)	Range	Number of patients
Growth (mm/year )	1.85 (0.88–2.93)	− 1.92 to 9.09	167
*d*_2*D*_ (mm)	43 (37–48)	29–59	167
*d*_3*D*_ (mm)	38 (34–45)	23–58	167
*Vpart,*30 (ml)	31 (24–44)	15–72	159
*Vpart,*40 (ml)	40 (31–55)	18–96	149
*Vpart,*50 (ml)	46 (37–65)	21–121	120
*Vpart,*60 (ml)	60 (41–78)	24–146	78
*Vpart,*70 (ml)	77 (56–96)	28–169	41
*K*1*AA* (m^−1^)	59 (52–67)	38–89	167
*K*2*AA* (m^−1^)	8 (6–12)	0–20	167
*K*1*LN* (−)	0.42 (0.38–0.46)	0.25–0.54	167
*K*2*LN* (−)	0.17 (0.15–0.19)	0.09–0.23	167
*GAA* (m^−2^)	34 (32–38)	22–47	167
*MAA* (m^−1^)	369 (246–526)	− 133 to 1115	167
*GLN* (−)	0.021 (0.018–0.024)	0.006–0.034	167
*MLN* (−)	11.5 (10.1–13.7)	6.3–19.5	167
*D* (10^−5^ Pa^−1^)	0.45 (0.29–0.6)	0.15–1.36	122
*C*_30_ (10^−5^ ml/Pa)	14 (10–18.8)	3.9–62	118
*C*_40_ (10^−5^ ml/Pa)	17.9 (12.5–25.1)	5–78.9	111
*C*_50_ (10^−5^ ml/Pa)	21.1 (15.1–29.7)	6.1–93.6	94
*C*_60_ (10^−5^ ml/Pa)	25.5 (17.9–34)	6.7–93	63
*C*_70_ (10^−5^ ml/Pa)	31.1 (22.2–42.8)	10.2–105	37

**Fig. 2:**
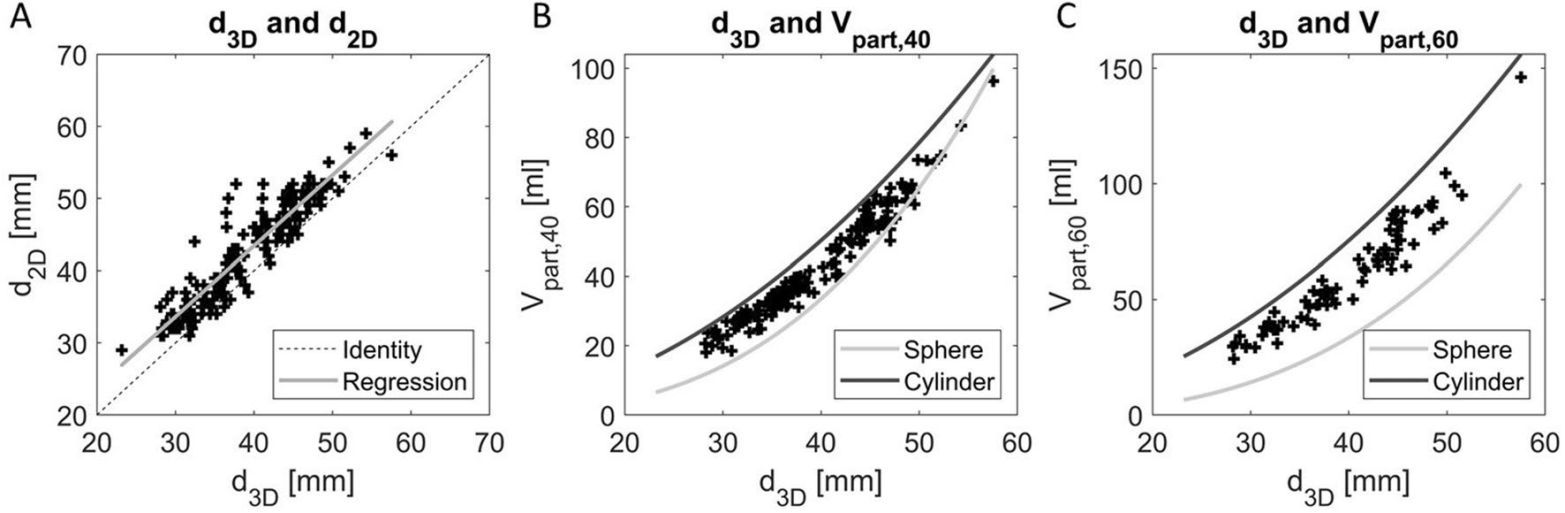
Relationship between the 3D-ultrasound based maximal diameter (d_3D_) and **A** the 2D- ultrasound based diameter (d_2D_), **B** partial volume in a 40 mm long region (V_part,40_), and **C** partial volume in a 60 mm long region (V_part,60_). The volumes of a sphere and cylinder with diameter d_3D_ are indicated in grey and black, respectively.

The obtained values of the growth, geometrical, and mechanical parameters (Table 2) show a large variety in growth rates (− 1.92 to 9.09 mm/year) and distensibility values (0.15–1.36 × 10^−5^ Pa^−1^) for patients with a variety of AAA diameters (29–59 mm). The values of *d*_3*D*_ are structurally lower than for *d*_2*D*_, with a patient-wise difference of 3.2[1.6–4.7] mm (Fig. 2).

From the 35 patients with a CT scan within 2 months of a 3D + t echogram, three patients had US data with very poor quality, from which the location of the AAA wall could not be determined by visual inspection. The automatic segmentation of one of the remaining 32 US data sets was hindered by bright reflection of the thrombus, which decreased the visibility of the AAA wall. Finally, for two patients, the matching between the US and CT geometries was ambiguous because of multiple regions in the CT-based segmentation resembling the region visible in US, leaving 29 patients for comparison.

### Comparison of the Geometry to CT

**Fig. 3:**
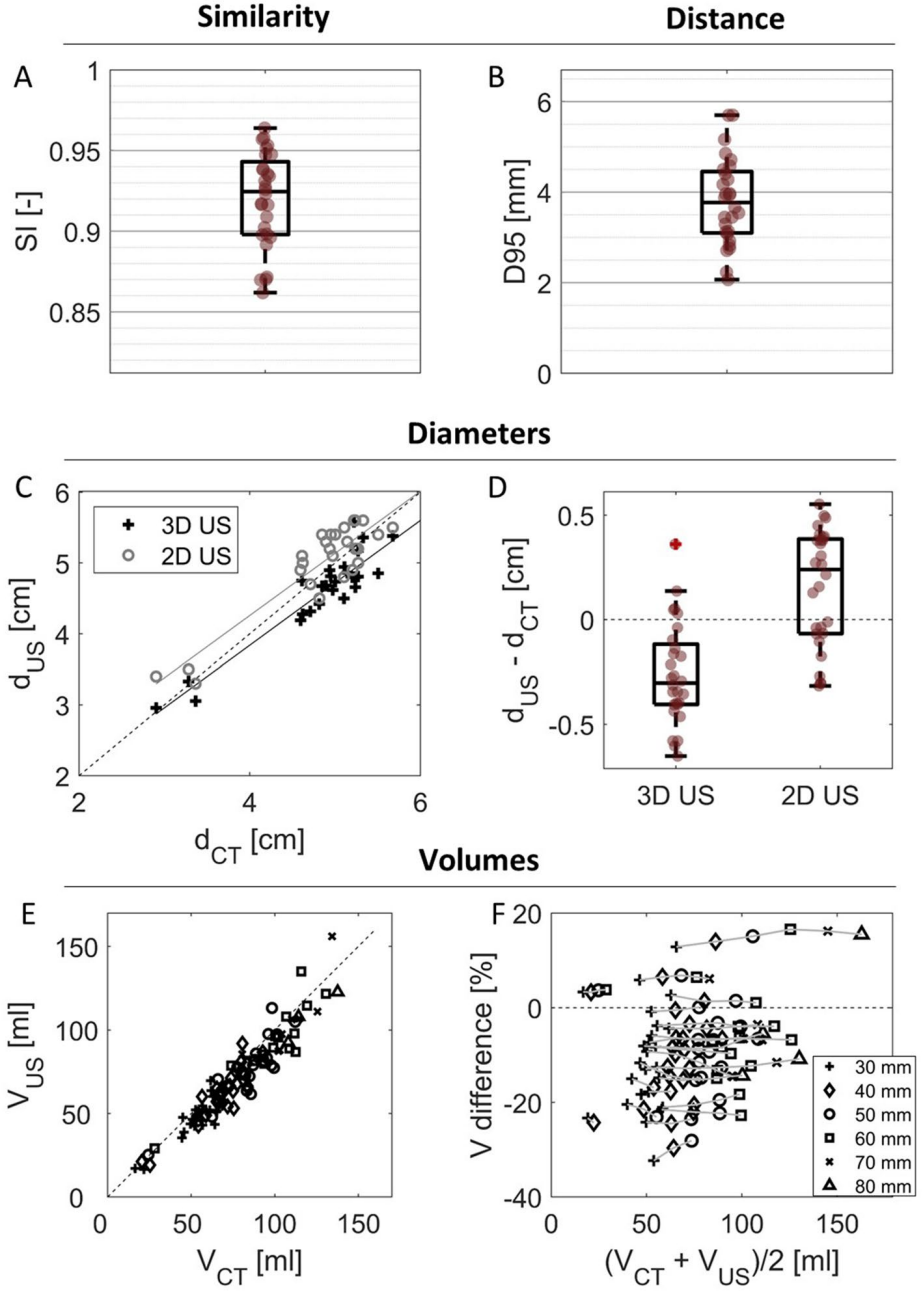
Comparison of the geometry based on ultrasound (US) and computed tomography (CT). **A**, **B** Box-and-whiskersplots of the similarity index (SI) and 95-percentile distance (D95) with individual data points in red. **C** The diameters from 2D and 3D US (d_US_) plotted against diameters from CT (d_CT_), with linear fits shown in grey and black, respectively. **D** Box-and-whiskersplots of the differences in diameters, with individual data points in red. **E** Volumes from US (V_US_) and CT (V_CT_) for regions of different lengths plotted against each other. **F** Percentual difference between volumes from US and CT, with volumes from the same patient connected with grey lines. Identity lines (**C**, **E**) and zero-lines (**D**, **F**) are added for visual aid (dotted lines)

The good correspondence between the CT and US geometries is seen from the high SI (0.92 [0.90–0.94]) and low *D95* (3.8 [3.1–4.5] mm) (Fig. 3A, B). Local evaluation shows that the SI is generally lower towards the proximal and distal end of the geometry [39]. Good correspondence is also seen between both *d*_*CT*_ and *d*_3*D*_, and *d*_*CT*_ and *d*_2*D*_, with a RMSE of 3.5 mm and 3.1 mm, respectively (Fig. 3C, D). The 3D US-based diameter usually underestimates the CT diameter, while the 2D US-based diameter overestimates the diameter. The range in differences is of comparable magnitude and even slightly smaller for 3D US, with an IQR of 2.9 mm and 4.5 mm for 3D and 2D US, respectively.

The volumes determined from the US images are generally slightly lower than those determined from CT (Fig. 3E, F). Per patient, the percentual underestimation of the CT volume by US is mostly constant with increasing size of the partial volume region, with an average of 11[5–8] % for volumes of 30 mm long.

**Fig. 4:**
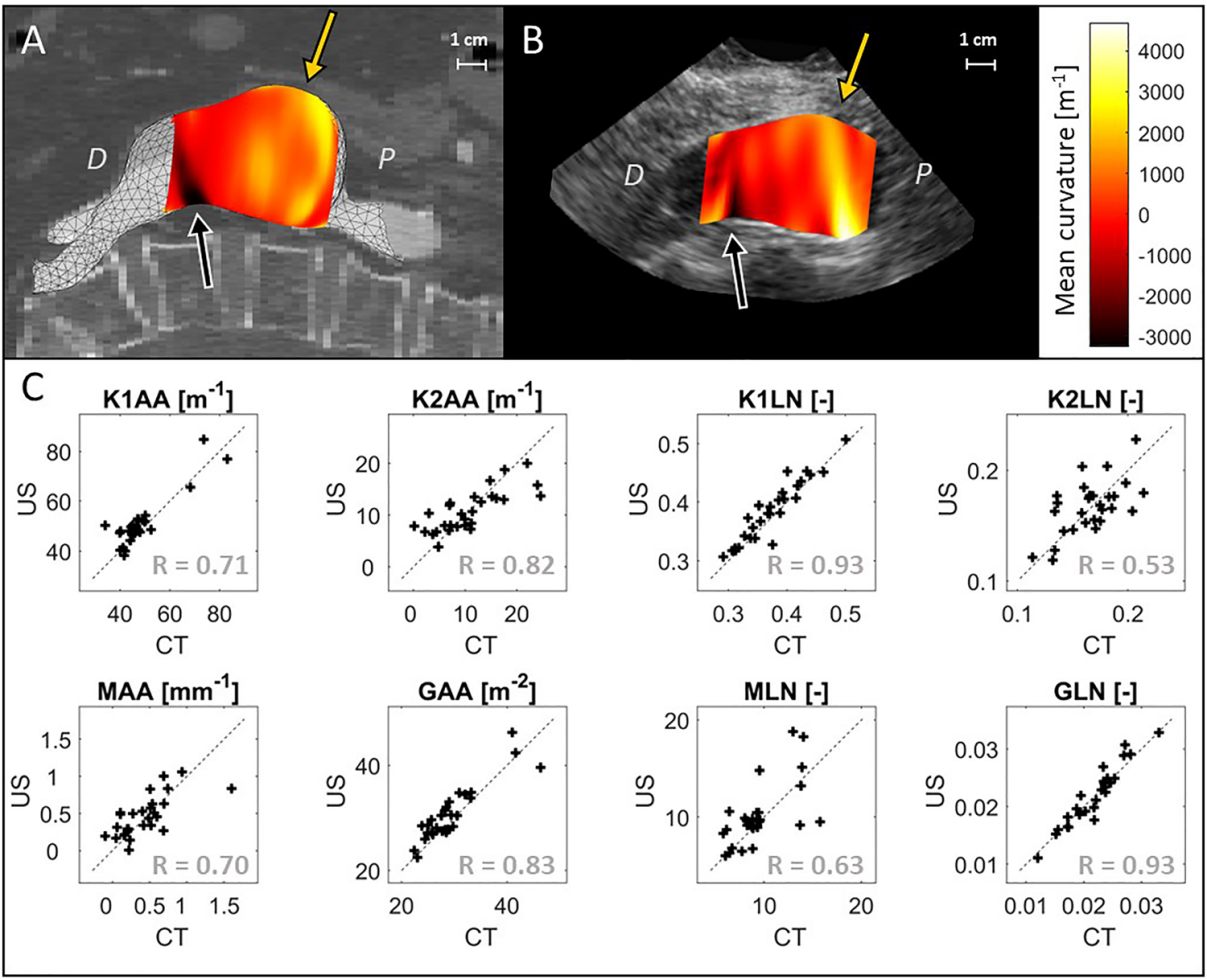
Comparison of the surface curvature of the AAA based on ultrasound (US) and computed tomography (CT). An example of **A** the CT-based curvature on the CT scan and **B** the US-based curvature on the US scan, with the yellow and black arrow indicating similar regions with high and low mean curvature, respectively. The proximal and distal side of the AAA are indicated with ’P’ and ’D’. **C** The similarity of eight curvature measures based on US and CT for 29 patients, in which the area-averages [X]AA and L2-norm [X]LN of the first and second principal curvatures K1 and K2, and of the mean and Gaussian curvatures K and G are shown. The Spearman’s R values are shown in grey

A typical example of the curvature derived from US and CT (Fig. 4A, B) shows similar curvature values and global patterns, with some small local differences. The CT-derived curvature parameters show moderate (*K*2*LN* with R = 0.53) to very strong (*K*1*LN* with R = 0.93) correlation with US-based curvature (Fig. 4C), with stronger correlations for parameters only dependent on *K1*.

### Correlation Between Determined Parameters

**Fig. 5:**
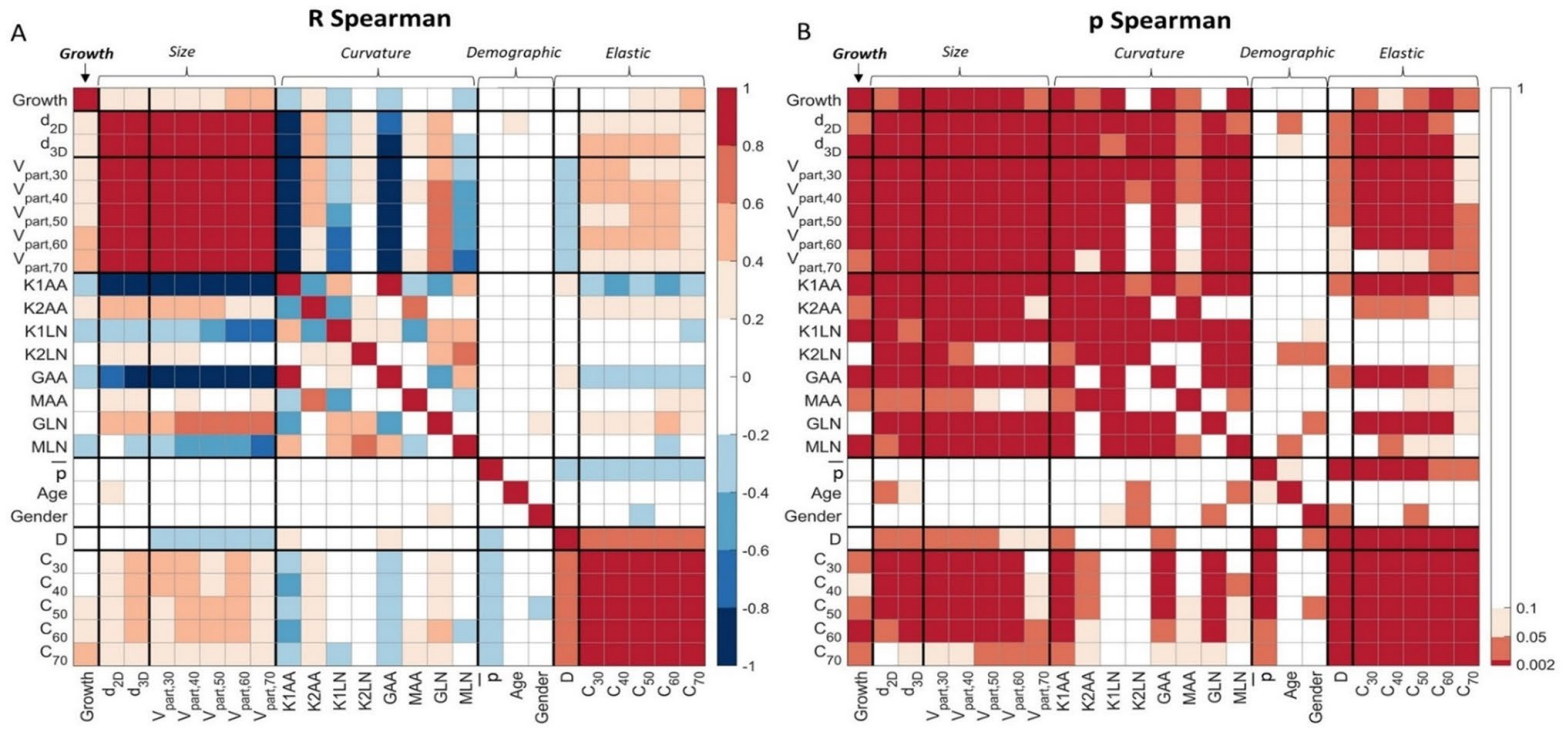
Spearman’s R (**A**) and correlation p-value (**B**) for all tested variables. In this, d_2D_ is the diameter from 2D ultrasound, d_3D_ the diameter perpendicular to the centerline from 3D ultrasound, V_part,L_ the volume in a L mm long region around maximal diameter. Furthermore, the area-averages [X]AA and L2-norm [X]LN of the first and second principal curvatures K1 and K2, and of the mean and Gaussian curvatures K and G are shown. p̅ is the mean arterial pressure, D the distensibility, and C_L_ is the compliance in a L mm long region around d_3D_

**Fig. 6:**
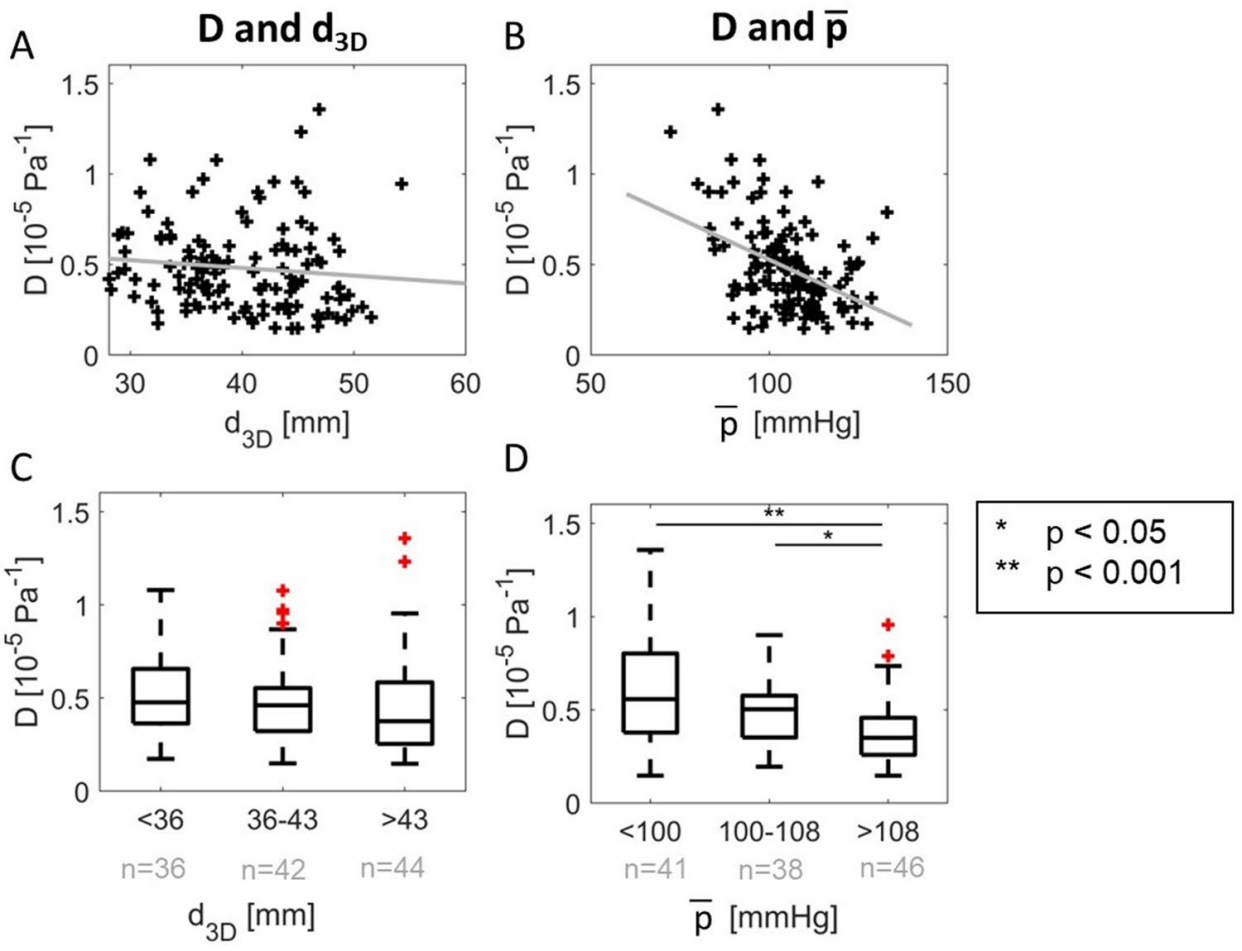
Correlation of the distensibility (D) with **A** the maximal diameter (d_3D_) and **B** mean arterial pressure (p̅), and comparison of D for equally sized groups based on **C** diameter and **D** pressure with a Wilcoxon rank sum test. The number of patients in each subgroup are indicated in grey

The Spearman’s R and p-values of the correlation between all investigated parameters (Fig. 5) show a high mutual correlation between *d*_2*D*_, *d*_3*D*_ and *V*_*part,L*_, with an R-value of ≥ 0.87 for the mutual correlations. The distensibility of the aorta is correlated to *p̅* (p* < *0.0001) and *d*_3*D*_ (p = 0.034). Splitting into three equal groups shows no significant differences for *d*_3*D*_ (p = 0.07 for small (< 36 mm) vs. large (> 43 mm) AAAs), and a significant decrease in distensibility between patients with highest *p̅,* and medium or low *p̅* (Fig. 6).

Figure 5 also shows that all size parameters, the compliance and curvature parameters *K1AA*, *K*1*LN*, *GAA*, and *MLN* have a statistically significant relation to upcoming growth. Some curvature parameters are however also strongly related to *d*_3*D*_, especially *K*1*AA* and *GAA* with R-values of − 0.90 and − 0.83, respectively. After correction for diameter, *K*1*LN*, *K*2*LN* and *MLN* are significantly correlated to growth with p = 0.0066, p = 0.0059 and p = 0.0114, respectively. This suggests the added value of curvature as a growth predictor.

### Linear Growth Model

**Fig. 7:**
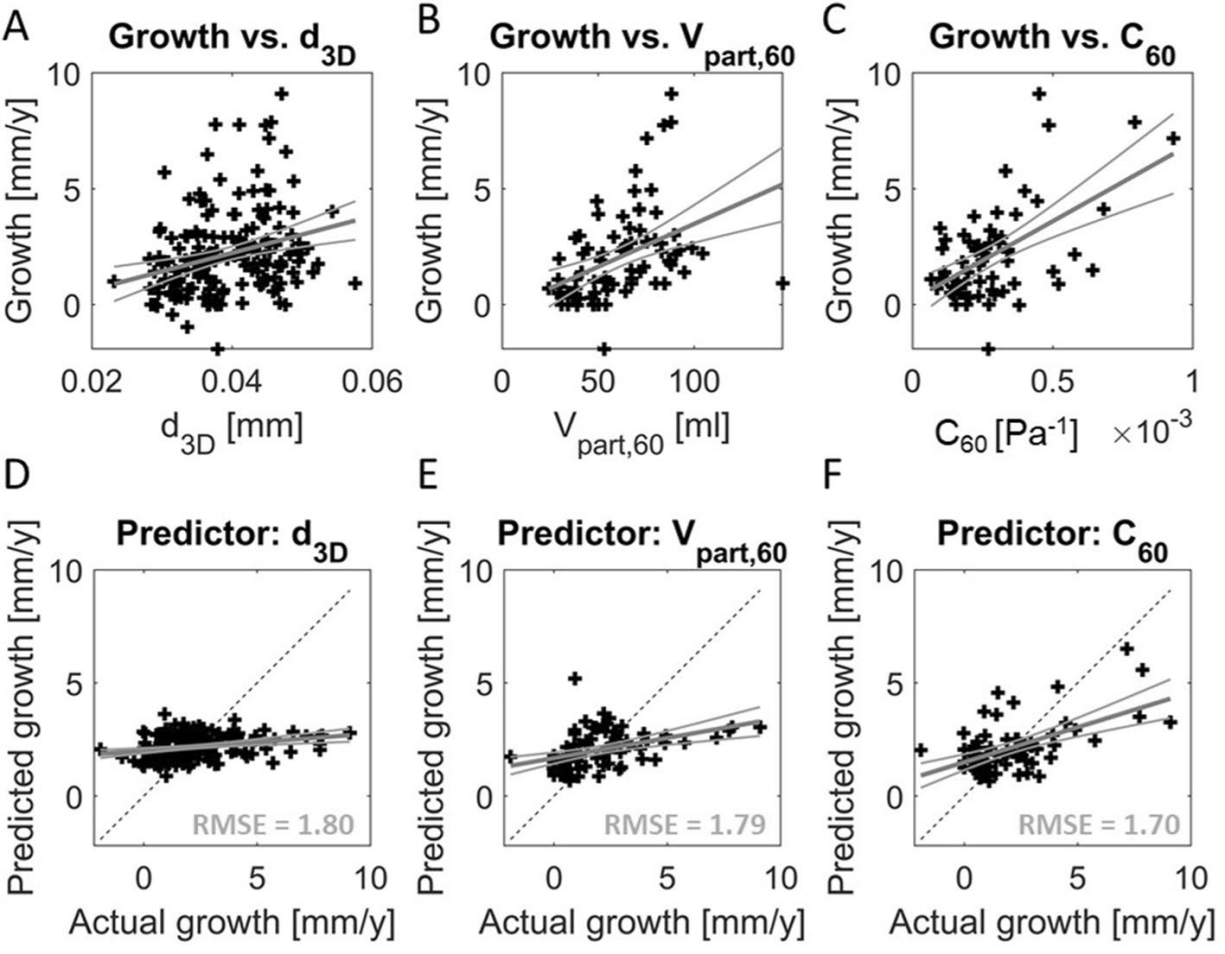
Relationship between the growth of the AAA and **A** aortic diameter (d_3D_) (N = 167), **B** volume in 60 mm (V_part,60_) (N = 78), and **C** compliance in 60 mm (C_60_) (N = 63). A linear fit between the two variables is shown in grey. The relationship between the actual growth, and prediction growth based on **D** d_3D_, **E** V_part,60_ and **F** C_60_. RMSE values between the actual and predicted growth are shown in grey

Stepwise linear regression indicated *C*_60_ as the best predictor for upcoming growth, despite calculation of *C*_60_ only being feasible for 63 out of 167 patients. The growth models based on *C*_60,_ *d*_3*D*_ and *V*_60_ (Fig. 7) show that both *d*_3*D*_ and* V*_60_ can explain only a small part of the variation in AAA growth, with the range in predicted growth much smaller than the actual growth: 0.9 to 3.6 mm/year versus − 1.9 to 9.1 mm/year, respectively, for *d*_3*D*_*.* With *C*_60_ as a predictor, for some patients with higher growth, the prediction is closer to the actual growth value, resulting in the lowest RMSE value.

## Discussion

In this study, we have shown that with 3D + t US, it is possible to determine both geometrical and mechanical parameters of the maximally dilated region of AAAs in a fully automated manner. Comparison between geometrical parameters derived from US and CT was performed, and correlation between the parameters and their relation to upcoming growth were determined.

The principal findings of this study are the following:

1. It is possible to automatically determine both geometrical and mechanical parameters of AAAs from 3D + t echography, with a success rate of 97% for the geometry, 73% for the distensibility, and 88% and 47% for determining a 30 and 60 mm long volume, respectively. The resulting geometries show good correspondence to CT geometries (median SI 0.92).
2. Large variations between patients are observed in mechanical parameters (*D* between 0.15 and 1.36 × 10^−5^ Pa^−1^), geometries and growth rates (− 1.9 to 9.1 mm/year).
3. The growth rate of the AAA is related to the diameter, volume, compliance and multiple curvature measures. The compliance of the AAA seems the best predictor for upcoming AAA growth.

### Performance of the Developed Tool

Despite the high success rate of the automatic segmentation, problems occurred for five patients because of poor lateral contrast (two patients), a very bendy shape of the AAA (one patient) and the segmentation going towards the lumen-thrombus interface (one patient) or the spine (one patient). In future studies, these problems could be corrected by making a few manual annotations, guiding the algorithm towards the correct structure (e.g. away from the thrombus, towards the vessel wall). The high similarity of the successful segmentations with CT data shows that 3D + t US with our automated processing tool provides a reliable method for determining the geometry of a AAA.

Determining the pulsatile motion of the AAA from 3D + t US is challenging, as the aorta only extended 0.6 [0.47–0.81] mm (d_3D_), while the average aortic center depth was 6.3 cm, resulting in a lower resolution in the US images because of high absorption. Furthermore, the lower temporal and spatial resolution of 3D + t US compared to 2D + t US will hinder the motion tracking further. Despite these challenges, the distensibility could be determined for 73% of the patients. In patients where this was not possible, visible inspection also showed less clear pulsations, showing the limits of temporal and spatial resolution of 3D + t US.

The assessment of the larger volumes was hindered by the lower visibility of the aortic wall towards the proximal and distal sides of the 3D volume, due to unfavorable angle between the incoming US signal and aortic wall. Despite this limited field of view, the shoulders of the aneurysm were visible in 126 out of 172 patients. This drawback of ultrasound could be overcome by making multiple proximal and distal 3D + t US images, and registering and fusing these images [40], leading to a larger part of the AAA being visible.

### Obtained Growth and Mechanical Parameters

As expected from previous research, large variations in growth rates and mechanical parameters were found in this study. Both could partially be explained by variety in AAA size (Figs. 6 and 7), as found in previous studies [3, 12, 15, 16, 21]. However, despite being statistically significant with p = 0.034 and p = 0.00013, there is a lot of dispersion in the data (R = − 0.19 and R = 0.29, respectively). The distensibility values of 0.45 (0.29–0.6) × 10^−5^ Pa^−1^ (median[IQR]) found in our study are in correspondence to previous studies by Ganten et al. [41] (mean: 0.6, SD: 0.5 × 10^−5^ Pa^−1^) and Zha et al. [42] (mean: 0.49, SD: 0.18 × 10^−5^ Pa^−1^) on ECG-gated CT, making 3D + t US a reliable and cost-effective method for determining distensibility, provided that the pulsation is visible in these images.

Part of the variation in distensibility between patients can be explained by differences in their maximal AAA diameter and *p̅*, which both have negative correlation with distensibility. These relationships were also found by Wilson et al. [43] from 2D ultrasound tracking, who explained that the loss of elasticity is related to the loss in elastin content.

The difference between *d*_2*D*_ and *d*_3*D*_, with *d*_2*D*_ being on average 3.2 mm higher than *d*_3*D*_, can partially be explained by a difference in definition. In the 2D US measurements performed for the clinical follow-up, the inner-to-outer wall diameter is measured, while the automatic segmentation segments the inner vessel wall. The aortic wall thickness is around 0.5–4 mm (median:1.5 mm) [44], whereas the rest of the difference might be explained by the wall thickness appearing a bit larger in the US images than it actually is because of the strong reflection of the vessel wall. Furthermore, there could also be problems with positioning of the US probe in 2D US measurements, which has led to previous calls for the use of 3D-US based diameter measurements [45].

### Growth Prediction

The predictive value of *C*_60_ for upcoming growth can partially be explained by the dependence of *C*_60_ on *V*_60_, as the relationship between growth and size of the AAA is well known [3, 15, 16]. Additionally, studies on biochemical markers have demonstrated a positive relationship between serum elastin peptides (SEP) and aortic distensibility [46], as well as between SEP and aneurysm growth [47]. This suggests that the aortic distensibility reflects modification of the aortic wall composition. This modification is expected to affect the AAA growth, through changes in local wall stresses and strains [48].

### Limitations

A first important limitation to this study is that only the geometrical parameters of the AAA could be validated, while no ground truth data was available for the mechanical parameters. For this, future ex vivo validation studies, for example in a mock-loop circulation setup could be performed, where the obtained stiffness could be verified with tensile testing.

Another limitation is that only linear growth rates were investigated in this study. The limited accuracy of 2D US-based diameter measurements, which is generally estimated at 2 mm [49, 50], prohibited the analysis of the pattern of the growth, while large differences in growth pattern between patients have been observed in previous studies with CT [51].

### Future Work

To further explore relationships between the investigated parameters, machine learning-based approaches can be of added value, capturing relationships that are missed by the linear regression models used in this study. Recent studies have shown that machine learning based on AAA geometry, combined with hemodynamics [19] or biomechanics [52], can predict the growth rate of AAAs. Repeating these studies with US-based AAA characteristics would be a logical next step in making these methods more feasible for follow-up AAA care.

Another next step in this research involves utilizing this automated and non-invasive method in a prospective longitudinal study using 3D + t US. This allows tracking changes in volume, distensibility and curvature values over time, providing better understanding of the development of these parameters.

Furthermore, local mechanical analysis of the AAA would be of interest, as rupture is a local phenomenon. For this, the displacement of the AAA should be determined locally, which is challenging with current state-of-the-art 3D + t US imaging because of the poor lateral and elevational resolution. The use of multi-perspective ultrasound, as well as the use of radio frequency data, can improve the estimation of displacements [53], allowing for local material property analysis of the AAA.

### Conclusion

In this work, we have shown the use of 3D + t US imaging to automatically determine mechanical and geometrical parameters of the region of maximal AAA diameter, which are related to AAA growth. The obtained geometries closely resemble CT-based geometries. Relationships of diameter, volume, curvature and compliance to growth have been shown, with the latter showing to be the best predictor for upcoming growth. This is a step towards a more insightful and patient-specific characterization of AAAs, that in the future will help to improve clinical decision making.
